# Variant-specific antibody profiling for tracking SARS-CoV-2 variant infections in children and adolescents

**DOI:** 10.3389/fimmu.2024.1434291

**Published:** 2024-08-27

**Authors:** Daniela Kuthning, Dina Raafat, Silva Holtfreter, Jana Gramenz, Nico Wittmann, Barbara M. Bröker, Almut Meyer-Bahlburg

**Affiliations:** ^1^ Pediatric Rheumatology, Department of Pediatric and Adolescent Medicine, University Medicine Greifswald, Greifswald, Germany; ^2^ Institute of Immunology, University Medicine Greifswald, Greifswald, Germany; ^3^ Department of Microbiology and Immunology, Faculty of Pharmacy, Alexandria University, Alexandria, Egypt

**Keywords:** SARS-CoV-2, children, adolescents, variants of concern, silent infections, antibody, spike S1

## Abstract

Monitoring the seroprevalence of SARS-CoV-2 in children and adolescents can provide valuable information for effective SARS-CoV-2 surveillance, and thus guide vaccination strategies. In this study, we quantified antibodies against the spike S1 domains of several SARS-CoV-2 variants (wild-type, Alpha, Delta, and Omicron variants) as well as endemic human coronaviruses (HCoVs) in 1,309 children and adolescents screened between December 2020 and March 2023. Their antibody binding profiles were compared with those of 22 pre-pandemic samples from children and adolescents using an in-house Luminex^®^-based Corona Array (CA). The primary objectives of this study were to (i) monitor SARS-CoV-2-specific antibodies in children and adolescents, (ii) evaluate whether the S1-specific antibody response can identify the infecting variant of concern (VoC), (iii) estimate the prevalence of silent infections, and (iv) test whether vaccination or infection with SARS-CoV-2 induce HCoV cross-reactive antibodies. Both SARS-CoV-2 infection and vaccination induced a robust antibody response against the S1 domain of WT and VoCs in children and adolescents. Antibodies specific for the S1 domain were able to distinguish between SARS-CoV-2 VoCs in infected children. The serologically identified VoC was typically the predominant VoC at the time of infection. Furthermore, our highly sensitive CA identified more silent SARS-CoV-2 infections than a commercial ELISA (12.1% vs. 6.3%, respectively), and provided insights into the infecting VoC. Seroconversion to endemic HCoVs occurred in early childhood, and vaccination or infection with SARS-CoV-2 did not induce HCoV S1 cross-reactive antibodies. In conclusion, the antibody response to the S1 domain of the spike protein of SARS-CoV-2 is highly specific, providing information about the infecting VoC and revealing clinically silent infections.

## 1 Introduction

The global spread of the severe acute respiratory syndrome coronavirus 2 (SARS-CoV-2) and the continuous emergence of new variants of concern (VoCs) have resulted in over 676 million infections and a death toll of approximately 6.9 million (as of 10/03/23) due to the associated coronavirus disease 2019 (COVID-19) (1). In Northern Germany, there have been four epidemic waves of COVID-19, caused respectively by the original SARS-CoV-2 strain Wu01 (March – December 2020), the Alpha variant (December 2020 – June 2021), the Delta variant (June 2021 – January 2022), and the Omicron variants BA.1, BA.2, BA.4 and BA.5 (from January 2022) (2).

Epidemiological data indicate that children are less prone to develop COVID-19 upon exposure to SARS-CoV-2 and, when infected, the symptoms are less severe than those in adults (3, 4). These milder disease courses are attributed to several factors, including earlier and more rapid type 1 interferon responses, increased cytokine production and differences in immune cell numbers (5, 6).

Asymptomatic, i.e. silent infections (SI) represent a significant potential driver of SARS-CoV-2 epidemics, as they can facilitate uncontrollable transmission. The role of asymptomatic children in viral transmission was a highly debated topic at the beginning of the pandemic. Recent studies, however, indicate that children are less frequently identified as index cases than adults in household and school settings (3), and that asymptomatic SARS-CoV-2-infected children are less likely to transmit the virus to other household members than symptomatic individuals (7). Knowledge of SI prevalence is essential for more accurately estimating transmission dynamics, improving epidemiological modelling, and guiding effective public health measures (8, 9).

Monitoring SI relies on the detection of viral RNA (PCR) or proteins (lateral flow tests) in swab samples, or on serological assays (e.g. ELISA). In the early stages of the pandemic, PCR testing was primarily focused on symptomatic cases and their direct contacts (10). Subsequently, the advent of lateral flow tests for public use in March 2021 facilitated mass testing, particularly in educational institutions such as schools and kindergartens (11). Serological assays are frequently employed in surveillance studies to ascertain the prevalence of SI (12, 13). Seroconversion to spike S1 protein is more common than to nucleocapsid (NC) protein (14), and serves as a marker for previous infection in unvaccinated cohorts.

The infection with the SARS-CoV-2 virus results in the formation of antibodies against a number of antigens, including the Spike (S) protein and NC. The Spike protein is composed of two domains, S1 and S2, which are responsible for mediating receptor binding and virus-host membrane fusion, respectively (15). The S2 domain is highly conserved amongst SARS-CoV-2 and closely related coronaviruses, and shares numerous antibody epitopes. Neutralizing antibodies primarily target the more variable S1 domain. The human immune response against SARS-CoV-2 drives viral evolution, leading to the emergence and global spread of VoCs with Spike variants that are less well recognized by vaccine-induced antibodies (16–18). The majority of amino acid exchanges are located in the spike S1 domain (18). Consequently, vaccine-induced antibodies and also therapeutic neutralizing antibodies are largely ineffective against the Omicron variant.

In addition to SARS-CoV-2, children frequently contract the closely related common cold human coronaviruses (HCoVs), including the alphacoronaviruses 229E and NL63 and the betacoronaviruses HKU1 and OC43. The vast majority of children experience their first HCoV infection at an early age and are subsequently re-exposed throughout their lives (19–21). Due to conserved epitopes, pre-existing antibodies from prior infections with HCoVs can cross-react with SARS-CoV-2 S and NC proteins (20, 22, 23). Nevertheless, it seems that seasonal HCoV infection does not confer cross-protection against SARS-CoV-2 infection (20, 22, 23).

Here, a total of 1,309 children aged six months to 17 years were sampled in North-Eastern Germany between December 2020 and March 2023. SARS-CoV-2 antibody profiling was performed using both commercial ELISA-based assays and an in-house Luminex^®^-based approach. The Luminex^®^-based Corona Array (CA) demonstrated greater sensitivity, resulting in an under-ascertainment rate of 12.1% among children and adolescents. Moreover, antibody profiles against the spike S1 domain from wild-type (WT) and VoCs were highly discriminatory and reflected the kinetics of VoC waves in Northern Germany. This approach enabled us to attribute the identified SI to the infecting variant with the highest probability. Finally, seroconversion to endemic HCoVs occurred at an early age. With regard to the variable S1 domain, COVID-19 vaccination or SARS-CoV-2 infection did not induce HCoV-cross-reactive antibodies.

## 2 Methods

### 2.1 Patient recruitment

Serum or plasma samples were collected from 22 pre-pandemic children and adolescents (aged 4-17 years) as well as from 1,309 children and adolescents (aged six months to 17 years; COVIDKID cohort) attending medical care in one of six participating hospitals and two private pediatric practices in North-Eastern Germany between December 2020 and March 2023. Repeated participation was permitted after a minimum interval of two months. Additionally, the families of the participants were requested to complete a questionnaire concerning the children’s SARS-CoV-2-vaccination status, previous SARS-CoV-2 infections, demographic data and socioeconomic background.

The study was approved by the Ethics Committee of the University Medicine Greifswald (BB188/20, and its amendment BB188/20a; BB 014/18) and entered in the German Clinical Trial Register on 09/03/2021 (Trial ID: DRKS00024635; https://drks.de/search/de/trial/DRKS00024635). All research was conducted in accordance with the tenets of the Declaration of Helsinki. All requirements of data protection and confidentiality were fully respected.

The study period was divided into distinct SARS-CoV-2 waves based on publicly available data from CoMV-Gen (24). Turning points of a new dominant VoC were interpolated from weekly proportions of challenging variants (interpolated proportion >50% for respective VoC). This resulted in the delineation of the following pandemic waves: I) Alpha wave (10/12/2020 – 19/06/2021), II) Delta wave (20/06/2021 – 05/01/2022), BA.1 wave (06/01/2022 – 16/02/2022), BA.2 wave (17/02/2022 – 07/06/2022) and BA.5 wave (08/06/2022 – 13/03/2023).

### 2.2 ELISA for the detection of SARS-CoV-2-specific IgG antibodies

The presence of SARS-CoV-2-specific IgG antibodies against the spike S1 domain and the nucleocapsid protein (Anti-SARS-CoV-2-ELISA (IgG) using the Spike S1 protein from the wild type strain and Anti-SARS-CoV-2-NCP-ELISA, respectively) was determined using commercially available kits (EI 2606-9601 G, EI 2606-9601-2 G; EUROIMMUN, Lübeck, Germany) according to the manufacturer’s instructions. Antibodies were detected semi-quantitatively and the results were interpreted as recommended by the manufacturer: positive at a ratio ≥1.1; negative at a ratio < 0.8; borderline at a ratio between 0.8 and 1.1. In brief, serum or plasma samples were diluted 1:101, and the levels of IgG antibodies against S1 or NC were analyzed using an Immunomat (Virion/Serion, Würzburg, Germany) or Tecan infinite M200 Pro microplate reader (Tecan Group Ltd., Männedorf, Switzerland), respectively.

### 2.3 12-plex Corona Array for the detection of SARS-CoV-2 and HCoV-specific IgG antibodies

The Corona Array (CA) is an in-house bead-based 12-plex suspension array based on the xMAP^®^ technology (Luminex^®^, Austin, USA) (25). The CA was designed for the simultaneous analysis of antibodies against different recombinant coronavirus antigens. Twelve recombinant proteins were procured from Sino Biological Europe GmbH (Eschborn, Germany), comprising seven recombinant His-tagged proteins/protein subunits of SARS-CoV-2; four recombinant spike S1 proteins of the endemic HCoVs, and the recall antigen tetanus toxoid (**Table 1**). The proteins were covalently coupled to MagPlex^®^ magnetic microspheres at a concentration of 100 µg per 1.25 × 10^7^ beads. The coupling efficiency (coupling factor) was determined via the His-tag as previously described in detail (26).

**Table 1:** Recombinant proteins used in this study for the construction of the CA.

	Recombinant protein/protein domain*	Abbreviation	Company	Catalog Number
SARS-CoV-2 (2019-nCoV)				
**1**	SARS-CoV-2 (2019-nCoV) Spike S1	S1_WT	Sino Biological	40591-V08H
**2**	SARS-CoV-2 (2019-nCoV) Nucleocapsid	NC_WT	Sino Biological	40588-V08B
**3**	SARS-CoV-2 (2019-nCoV) Spike S1 (Alpha variant)	S1_Alpha	Sino Biological	40591-V08H12
**4**	S1 Delta (B.1.617.2)	S1_Delta	Sino Biological	40591-V08H23
**5**	S1 Omicron (B.1.1.529)	S1_ BA.1	Sino Biological	40591-V08H41
**6**	S1 Omicron (BA.2)	S1_ BA.2	Sino Biological	40591-V08H43
**7**	S1 Omicron (BA.4/BA.5)	S1BA.4/5	Sino Biological	40591-V08H46
Human coronaviruses				
**8**	Human coronavirus (HCoV-229E) Spike/S1 Protein	S1_229E	Sino Biological	40601-V08H
**9**	Human coronavirus (HCoV-HKU1) Spike/S1 Protein	S1_HKU1	Sino Biological	40021-V08H
**10**	Human coronavirus (HCoV-NL63) Spike/S1 Protein	S1_NL63	Sino Biological	40600-V08H
**11**	Human coronavirus (HCoV-OC43) Spike/S1 Protein	S1_OC43	Sino Biological	40607-V08H1
Recall antigen				
**12**	Tetanus Toxoid	TT	Sigma Aldrich	582231

*all recombinant proteins/protein domains, except for the tetanus toxoid, were His-tagged.

The CA was performed with serum or plasma samples as previously described (26, 27). The plasma dilution was optimized based on 7-point dilution series (1:20 to 1:312,500) of 19 representative plasma samples of various anti-S1_WT IgG levels as determined by a commercial S1 IgG ELISA, in order to be able to detect both low and high antibody levels in a single measurement (**Supplementary Figure 1**). Based on this pretest, we selected a sample dilution of 1:10,000 to ensure a reliable detection of both high and low antibody levels. Plasma samples with a raw median fluorescence intensity (MFI) > 15.000 (n=46) were once more analyzed using 7-dilution series, to avoid saturation effects, and a single dilution within the linear range was selected for the analysis. A plasma pool (prepared from plasma samples of 13 donors with previous SARS-CoV-2 infection (COV^+^) and/or COVID-19 vaccination (VAC^+^)) was included on each plate for data normalization. The antibody binding was determined using the BioPlex 200 system (Bio-Rad Laboratories GmbH, Feldkirchen, Germany) with bead buffer serving as the blank. The following instrument settings were used: bead type: MagPlex beads, beads: 100 beads per region, sample timeout: 60 sec, sample volume: 80 μL, gate settings: 7,500–15,000 (BioPlex Manager 5.0 software; Bio-Rad Laboratories GmbH, Feldkirchen, Germany).

The relative IgG concentrations measured in the plasma pool were employed to normalize inter-plate variations. The coupling factor was utilized for correction between antigens. Serum samples were considered seropositive for SARS-CoV-2 when antibody binding to at least one S1 variant (WT or VoCs) was above the cut-off value of the respective antigen. Serum samples with only anti-NC antibodies above the cut-off value were not considered seropositive for SARS-CoV-2, as cross-reactivity to other coronaviruses can result in increased NC antibody levels.

### 2.4 Statistics

Statistical testing and data visualization were conducted using GraphPad Prism (v9) and R (v4.2.0; https://www.R-project.org/) in combination with the tidyverse package (v2.0.0) (28) and the patchwork package (v1.1.2; https://CRAN.R-project.org/package=patchwork). Cutoff values for the CA were defined based on MFI values of 22 pre-pandemic samples from children and calculated as mean + 5× standard deviation (SD).

The significance of differences between groups was tested by one-way analysis of variance (ANOVA; Kruskall-Wallis test) with *post-hoc* Dunn’s multiple-comparison test. Statistical significance was defined as a p-value <0.05. The correlation between the ELISA and CA was calculated using Spearman’s correlation coefficient.

## 3 Results

The dynamics of the antibody response to several SARS-CoV-2 variants were traced in 1,309 serum/plasma samples obtained from children and adolescents in North-Eastern Germany between December 2020 and March 2023 (median age: 10 years, range: 0.5-17 years). Serum samples from a pre-pandemic cohort of 22 individuals (pre-COVID; median age: 12 years; range 4-17 years) served as controls and for the calculation of the cut-off values (WT_S1: 50.4; WT_NC: 545.65; Alpha_S1: 74.58; Delta_S1: 63.46; BA.1_S1: 64.1; BA.2_S1: 60.3; BA.4/5_S1: 51.2). Questionnaires were employed to ascertain previous SARS-CoV-2 infections (COV^+^ or COV^-^) and/or COVID-19 vaccinations (VAC^+^ or VAC^-^). Among the 1,309 study subjects, 271 reported infection with SARS-CoV-2 (COV^+^) and 177 reported vaccination (VAC^+^) (**Table 2**). Serum/plasma samples were assigned to five distinct SARS-CoV-2 waves (Alpha, Delta, BA.1, BA.2, BA.5), based on the time point of study inclusion. A SARS-CoV-2 wave was defined as the period in which the respective VoC was responsible for more than 50% of the reported cases (**Table 2**).

**Table 2:** Patient characteristics of the COVIDKID cohort.

	Total (n=1,309)	Unvaccinated, VAC^-^ (n=1,107)	Vaccinated, VAC^+^ (n=177)	Unknown (n=25)
**Female, no. (%)**	668 (51.0)	549 (49.6)	104 (58.8)	15 (60.0)
**Age [years]** **median (range)**	10 (0.5 - 17)	9 (0.5 – 17)	15 (2 – 17)	13 (1 – 17)
Study inclusion during^1^				
**Alpha wave** **(10/12/2020 – 19/06/2021)**	496	481	4	11
**Delta wave** **(20/06/2021 – 05/01/2022)**	343	303	34	6
**BA.1 wave** **(06/01/2022 – 16/02/2022)**	99	62	37	–
**BA.2 wave** **(17/02/2022 – 07/06/2022)**	188	138	47	3
**BA.5 wave** **(08/06/2022 – 13/03/2023)**	183	123	55	5
SARS-CoV-2 infection status (COV)^2^				
**Undiagnosed (COV^-^)**	1009	876	120	13
**At least one diagnosis (COV^+^)**	271	210^3^	55	6
**Unknown**	29	21	2	6

^1^SARS-CoV-2 waves were defined as the period in which the respective VoC was responsible for more than 50% of the reported cases.

^2^Information about SARS-CoV-2 exposure at the time of study inclusion were evaluated from a questionnaire.

^3^including n=190 of COV^+^/VAC^-^ with a single diagnosis, of which n=143 with known date of diagnosis and analyzed for SARS-CoV-2 IgG profiles in **Figure 3**.

### 3.1 SARS-CoV-2 infection and vaccination induce high levels of S1-specific antibodies

**Figure 1:**
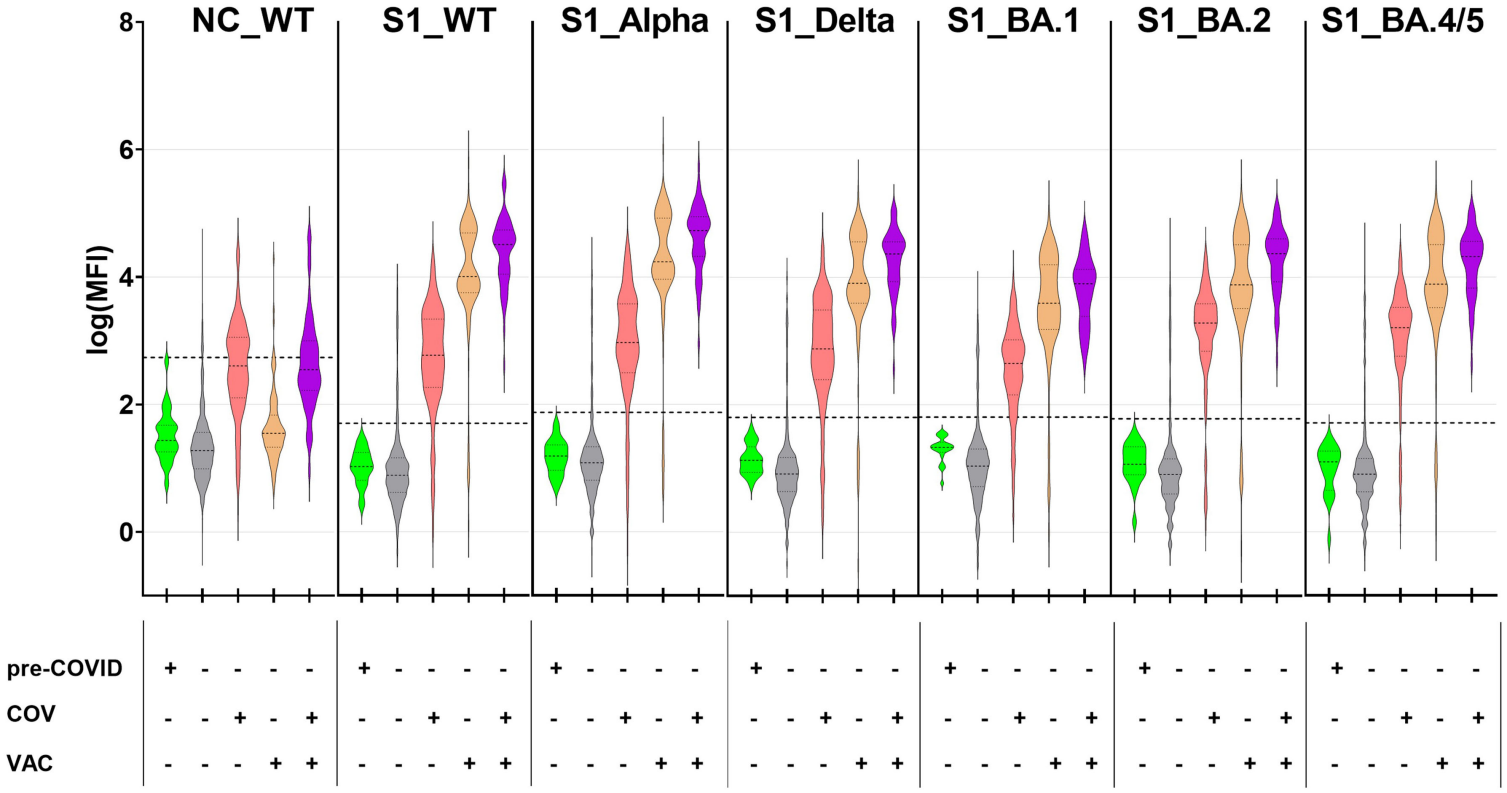
SARS-CoV-2-exposed children and adolescents exhibit a robust antibody response against SARS-CoV-2 antigens from wild-type SARS-CoV-2 and its VoCs. SARS-CoV-2-specific antibodies were quantified with the CA in 1261 pediatric samples with known SARS-CoV-2 vaccination and infection status as well as 22 pre-COVID samples, against nucleocapsid protein from SARS-CoV-2 wild-type (NC_WT), spike S1 domain from WT (S1_WT) and circulating VoCs (S1_Alpha, S1_Delta, S1_BA.1, S1_BA.2 and S1_BA.4/5). Data are displayed as log-transformed MFI values. Samples were categorized based on sampling time point (pre-COVID^+^/pre-COVID^-^), anamnestic SARS-CoV-2 infection (COV^+^/COV^-^) and COVID-19 vaccination status (VAC^+^/VAC^-^). Horizontal dotted lines represent the cut-off values for each of the tested antigens (mean (MFI) +5×SD). The dotted lines within the violin plots depict the median and the quartiles. Sub-cohorts: pre-COVID, n=22; COV^-^/VAC^-^, n=876; COV^+^/VAC^-^, n=210; COV^-^/VAC^+^, n= 120; COV^+^/VAC^+^, n=55.

The presence of SARS-CoV-2-specific serum antibodies (seroconversion) against NC and the S1 subunits of SARS-CoV-2 WT and VoCs was quantified using a bead-based in-house CA. The levels of anti-NC antibodies (NC_WT; **Figure 1**; panel 1) were found to be highest in children and adolescents who reported a previous COVID-19 infection (COV^+^; median log(MFI) 2.60). In contrast, the majority of vaccinated individuals without prior infection (COV^-^/VAC^+^; median log(MFI) 1.55), participants with neither infection nor vaccination (COV^-^/VAC^-^; median log(MFI) 1.28) and pre-COVID samples (median log(MFI) 1.44) lacked anti-NC antibodies. This is not unexpected, given that all SARS-CoV-2 vaccines approved for children and adolescents in Germany during the study period contained mRNA or DNA encoding the SARS-CoV-2 WT spike protein.

Vaccinated children and adolescents with (COV^+^/VAC^+^) or without infection (COV^-^/VAC^+^) mounted a robust serum IgG response against the S1 subunit of the SARS-CoV-2 spike protein. Their antibodies bound to the S1-domains of the SARS-CoV-2 strain Wu01 (wild-type, WT) and the five VoCs that circulated in the study region (**Figure 1**). The highest antibody levels were observed in children and adolescents who had experienced both infection and vaccination (COV^+^/VAC^+^), while infection alone induced significantly lower amounts of anti-S1 IgG serum antibodies (median log(MFI) 1.3- to 1.6-times lower in COV^+^/VAC^-^ than in COV^+^/VAC^+^ individuals; p < 0.001 in Kruskall-Wallis test for all S1 antigens; not shown). As anticipated, pre-COVID- and most COV^-^/VAC^-^ samples lacked anti-S1 antibodies. However, some COV^-^/VAC^-^ subjects exhibited high anti-S1 IgG levels, suggesting the possibility of silent SARS-CoV-2 infections.

Furthermore, the antibody binding patterns to the S1 from WT and circulating VoCs (Alpha, Delta, Omicron BA.1, BA.2, and BA.4/5) differed between vaccinated and infected subjects. In the vaccinated group (COV^-^/VAC^+^), antibody binding to the closely-related S1_WT and S1_Alpha (median log(MFI) 4.01 and 4.24, respectively) exhibited a tendency to be stronger than to S1 of the other VoCs (median log(MFI) 3.59 to 3.91). In the COV^+^/VAC^-^ group, the anti-S1 antibody binding patterns exhibited considerable inter-individual variability (median log(MFI) 2.7 to 3.3).

In conclusion, our CA data demonstrate that both vaccination and infection elicit robust anti-S1 antibody responses.

### 3.2 The Corona Array is more sensitive than a commercially available ELISA

**Figure 2:**
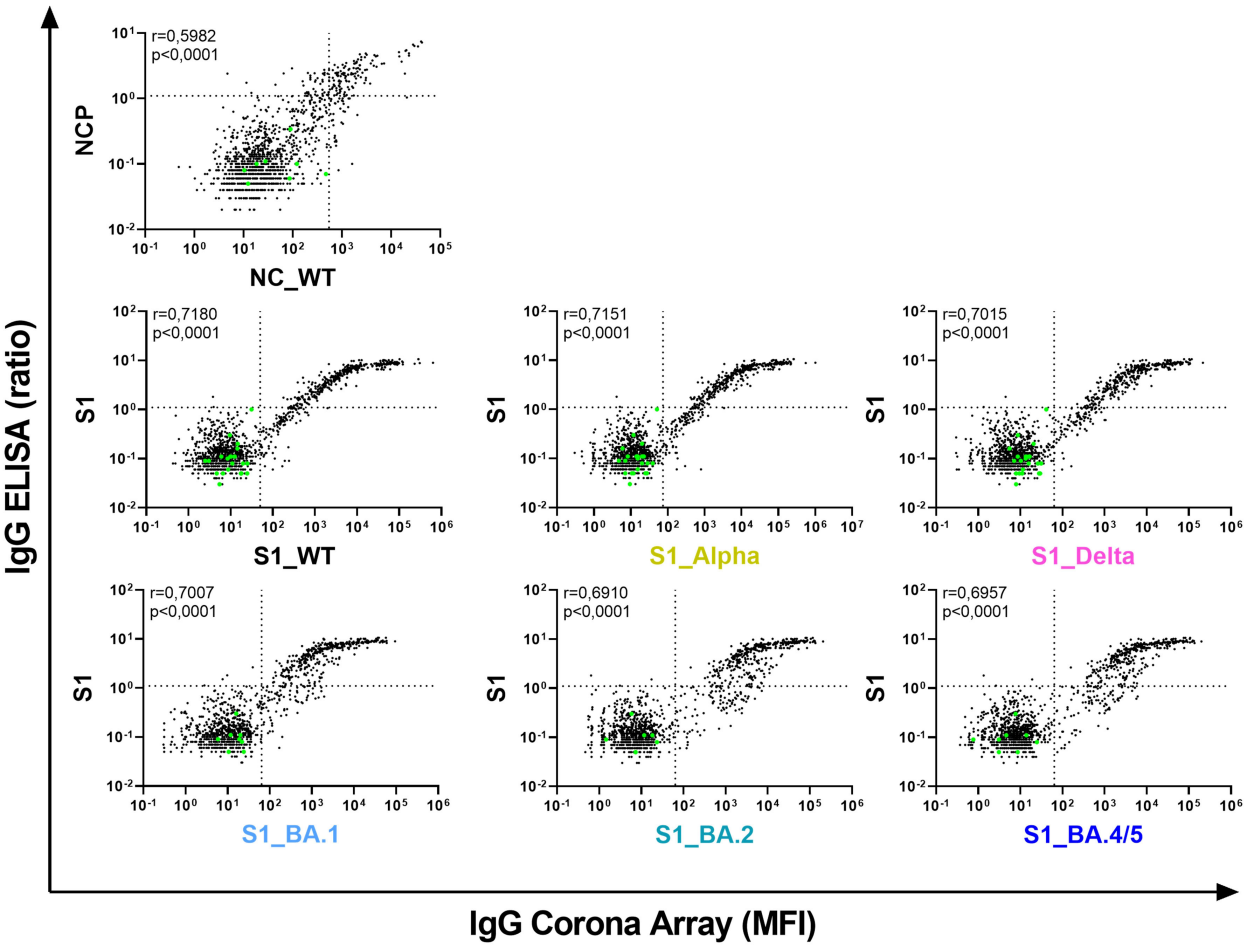
The Corona Array (CA) is more sensitive and covers a broader range than commercially available ELISAs. Serum IgG against nucleocapsid protein (NC) as well as S1 from SARS-CoV-2 WT and circulating VoCs (Alpha, Delta, BA.1, BA.2 and BA.4/5) were quantified in 1331 pediatric samples using the CA and are plotted on the x-axis. Pre-COVID samples (n=22; green dots) were also used for calculation of cut-off values for the CA (mean (MFI) +5×SD; indicated by vertical dotted lines). Serum IgG against nucleocapsid protein (NCP) and S1_WT were determined by commercial ELISA. Results are indicated as ratio of OD_sample_/OD_calibrator_, and are plotted on the y-axis. The cut-off values recommended by the manufacturer are indicated by horizontal dotted lines. Spearman´s correlation coefficient r is depicted. NC/NCP, nucleocapsid protein; MFI, median fluorescence intensity; S1, Spike S1 domain.

To assess the performance of our in-house CA, we compared its results (antibody binding to NC_WT, S1_WT, S1 of five circulating VoCs) with the data obtained with commercial ELISAs (anti-nucleocapsid (NCP) IgG and anti-S1 IgG). Both methods yielded concordant results, as evidenced by Spearman’s correlation coefficients between 0.69 and 0.71 for the individual S1 antigens (**Figure 2**). However, regarding the S1_WT antigen, the CA exhibited greater sensitivity than the commercial ELISA (lower right quadrant, with samples positive in CA but negative in ELISA assay), and its dynamic range was considerably larger, spanning 5-6 logs as compared to two logs for the ELISA (upper right quadrant).

### 3.3 Anti-NC antibodies alone are not a reliable indicator of SARS-CoV-2 infections

As both COVID-19 vaccination and SARS-CoV-2 infection induce anti-S1 antibody responses, we investigated whether seroconversion to NC could serve as a reliable marker for previous COVID-19 infection(s). Consequently, the CA (NC_WT) or ELISA (NCP) were used to determine the prevalence of anti-NC antibodies among unvaccinated children and adolescents (n=1,086), of whom n=210 had reported a SARS-CoV-2 infection (COV^+^/VAC^-^).

Less than 50% of individuals with anamnestic COVID-19 infections were seropositive for NC, regardless of the applied method (sensitivities of 0.44 and 0.49, for CA and ELISA respectively) (**Table 3**). This remained consistent throughout the pandemic waves, with the exception of the BA.5 wave, where only 29.9% of infected individuals showed a positive NC result with either method (data not shown). The specificity of both the CA and ELISA assays was 0.96. Consequently, a negative NC test does not rule out a SARS-CoV-2 infection, while a positive NC test reliably indicates a (silent) infection.

**Table 3:** SARS-CoV-2-specific anti-S1 and anti-nucleocapsid IgG-antibodies in a cohort of VAC^-^ children and adolescents (n=1,086).

	n	CA^1^				ELISA^2^			
		S1		NC_WT		S1		NCP	
		+	–	+	–	+	–	+	–
**COV^+3^**	210	195	15	92	118	129	81	103	107
**COV^-3^**	876	106	770	36	840	49	827	36	840
**PPV**		0.65		0.72		0.72		0.74	
**NPV**		0.98		0.88		0.91		0.89	
**Sensitivity**		0.93		0.44		0.61		0.49	
**Specificity**		0.88		0.96		0.94		0.96	

^1^cut-off values for CA were defined as mean (MFI) +5×SD from 22 pre-COVID samples.

^2^cut-off values for commercial anti-S1 and -NCP ELISAs were defined as ratio (OD_sample_/OD_calibrator_) ≥1.1, as per manufacturer´s instructions.

^3^self-reported SARS-CoV-2 infection status.

CA, Corona Array; NC/NCP, nucleocapsid protein; S1, Spike S1 domain; PPV, positive predictive value; NPV, negative predictive value; +, positive test result; -, negative test result.

In contrast, a positive CA result for at least one S1 antigen (WT or VoCs) identified a previous SARS-CoV-2 infection in unvaccinated children with a superior sensitivity of 0.93, compared to a sensitivity of 0.61 for the ELISA. Therefore, we used the CA-based detection of antibodies against at least one S1 of either WT or VoC as a marker for (silent) SARS-CoV-2 infection in the subsequent analyses.

### 3.4 The S1-specific antibody response discriminates between SARS-CoV-2 VoCs

We hypothesized that the immune system is capable of discriminating between the S1 allelic variants of the circulating VoCs, with the strongest antibody response directed against S1 of the infecting VoC. To test this hypothesis, we analyzed the antibody profiles in children and adolescents with a single SARS-CoV-2 infection with known date of diagnosis (COV^+^/VAC^-^; n=143) (**Table 2**). We then calculated the MFI ratio of antibody binding to each S1_VoC to that to S1_WT, which we subsequently refer to as the VoC-to-WT ratio.

**Figure 3:**
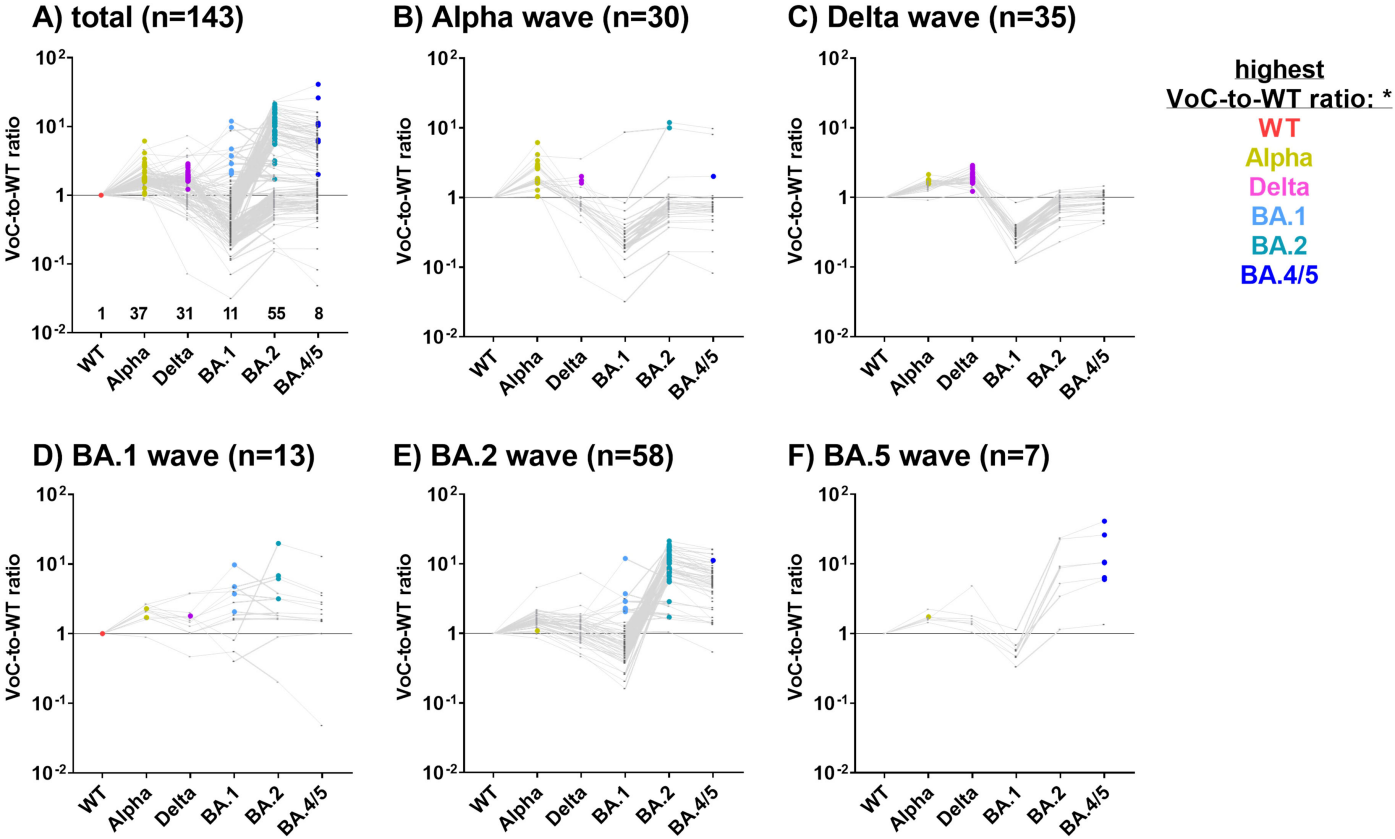
S1 variant-specific serological signatures can be used to identify the most probable infecting VoC. For 143 COV^+^/VAC^-^ SARS-CoV-2 CA_S1^+^ children and adolescents with a single SARS-CoV-2 diagnosis, antibody levels against S1 are depicted as ratios of MFI_VoC_ to MFI_WT_ (VoC-to-WT ratio) **(A)**. Subsequently, samples were assigned to the SARS-CoV-2 Alpha **(B)**, Delta **(C)**, BA.1 **(D)**, BA.2 **(E)** or BA.4/5 **(F)** waves based on the time of diagnosis. VoCs-to-WT ratios for each study subject are connected by a grey line. The VoC with the highest ratio is highlighted as a colored dot, likely reflecting the infecting VoC. Samples where all VoC-to-WT ratios were below 1 were assigned to the WT.

Samples were assigned to five distinct SARS-CoV-2 waves (Alpha, Delta, BA.1, BA.2 and BA.4/5) based on the time of diagnosis. In **Figure 3A**, the VoC with the highest ratio is highlighted as a colored dot for each individual, hinting towards the most probable infecting variant.

In subjects diagnosed with COVID-19 during the Alpha, Delta, BA.2 and BA.4/5 waves, the highest VoC-to-WT ratio was found to correspond closely with the dominant variant (**Figures 3B, C, E, F**). For example, during the Alpha wave, 24/30 study participants exhibited the strongest antibody binding to the S1 of the Alpha variant (**Figure 3B**, highlighted in gold), while three exhibited the strongest antibody binding to the Delta variant (pink dots). This does not contradict our assumption, as during each wave, there were also infections with non-dominant VoCs. Unexpectedly, three COV^+^ children who reported a SARS-CoV-2 infection during the Alpha wave exhibited the strongest binding ratios for Omicron variants BA.2 or BA.4/5, which were not yet present during the Alpha wave. These samples were obtained at a late stage of the pandemic, which might reflect additional Omicron infections that went undiagnosed (**Figure 3B**).

Of the 35 subjects diagnosed during the Delta wave, 27 (75%) exhibited the highest VoC-to-WT ratio for the Delta variant, while the remaining 8 samples demonstrated a strong reaction with the Alpha variant (**Figure 3C**). In individuals diagnosed during the BA.1 wave (n=13, **Figure 3D**), the results were inconclusive. The majority of samples diagnosed during the BA.2 and BA.5 waves presented with a BA.2- or BA.4/5-specific serological signature, respectively (**Figures 3E, F**).

In conclusion, antibody profiles against the VoC-S1 domains were highly discriminatory and reflected the kinetics of the VoC waves in Northern Germany.

### 3.5 Detection of silent SARS-CoV-2 infections

**Figure 4:**
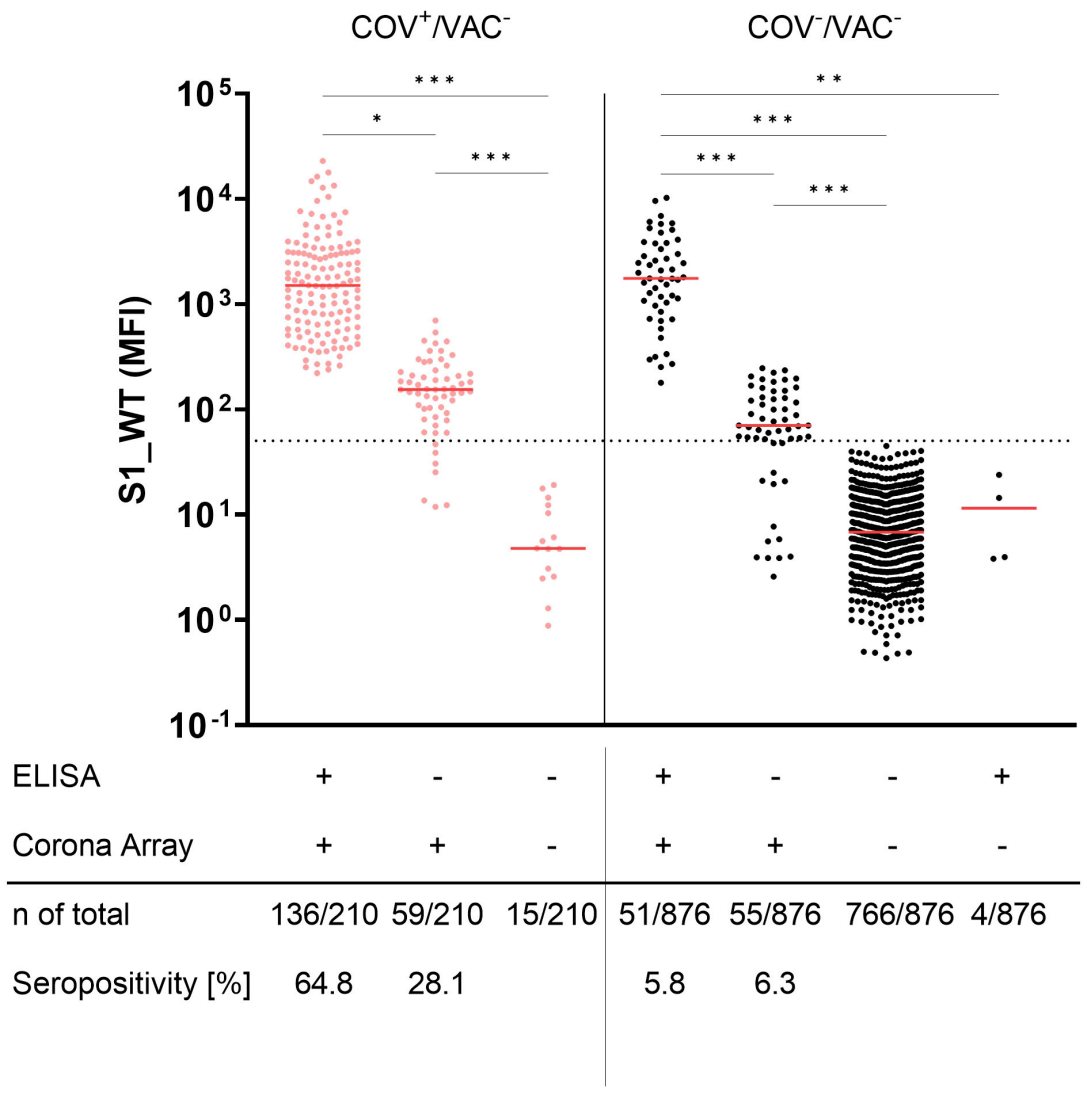
Corona Array (CA) detects SARS-CoV-2 infections with high sensitivity and unveils silent infections in 12.1% of anamnestically SARS-CoV2-naïve children and adolescents. Seroconversion to Spike S1 was determined by commercial ELISA and CA in pediatric COV^+^/VAC^-^ samples (red, left panel) and COV^-^/VAC^-^ samples (black, right panel). Samples were stratified by being positive in ELISA (S1_WT) and/or CA (S1 from WT or VoCs). ELISA^-^ but CA_S1^+^ samples from both groups show significantly lower levels of antibodies against S1_WT. The median is shown in red. The dashed line indicates the cut-off value for S1_WT. Significance between groups was tested by Kruskall-Wallis test and *post-hoc* Dunn´s correction. *, p<0.05; **, p < 0.01; ***, p<0.001.

Seroconversion to S1 is a commonly used marker for previous SARS-CoV-2 infections in unvaccinated individuals. However, the sensitivity of this approach depends on the method of detection. **Figure 4** depicts the antibody binding to S1_WT. While the commercial ELISA detected known SARS-CoV-2 infections in 136/210 unvaccinated children and adolescents (COV^+^/VAC^-^), CA was positive for at least one S1 allele in 195 of the 210 COV^+^/VAC^-^ participants (**Figure 4**, left panel). This results in 64.8% and 92.9% seropositivity for the ELISA and CA, respectively. The anti-S1_WT antibody levels in CA-positive (CA^+^) samples that were ELISA-negative (ELISA^-^) were found to be lower than in samples that were positive with both methods.

Using the same methods to identify unnoticed (silent) infections (SI), the commercial ELISA revealed anti-S1 antibodies in 6.3% (55 of 876 samples) of COV^-^/VAC^-^ children and adolescents. In contrast, the CA identified twice as many SI (106 of 876 samples; 12.1%). The positive samples that were missed by the ELISA (6.3%) exhibited lower S1-specific antibody levels against all tested S1 antigens than ELISA^+^ samples (**Figure 4**, right panel).

Looking at the whole COVIDKID cohort, a total of 301 of the 1,086 unvaccinated children had been exposed to SARS-CoV-2 based on the CA-S1 results. Of these, only 210 cases were diagnosed (COV^+^/VAC^-^). This results in a 1.43-fold higher SARS-CoV-2 exposure rate than reported.

### 3.6 Antibody signatures specific to the S1-variant provide insights into the contact variant in children with silent infections

**Figure 5:**
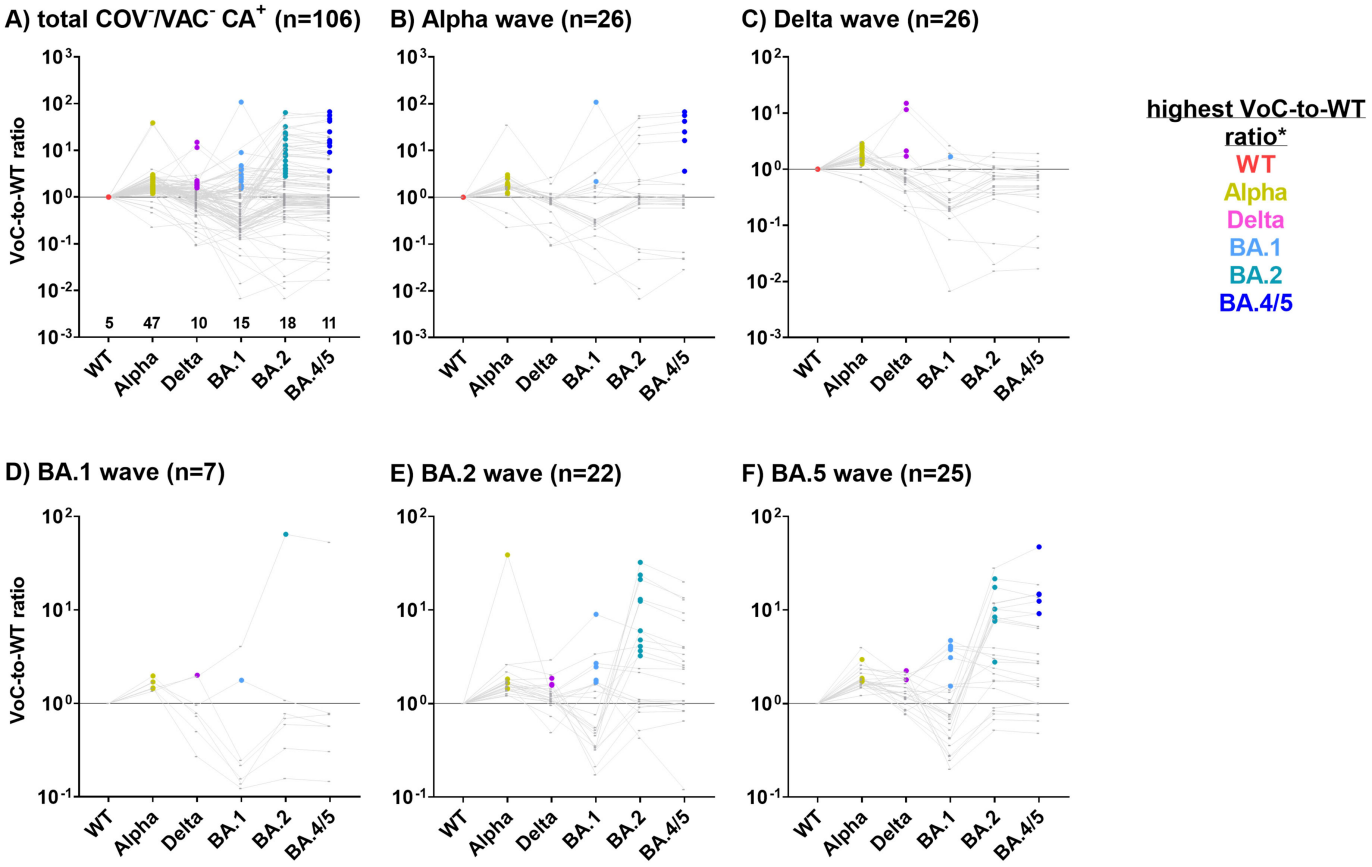
Children and adolescents silently infected with SARS-CoV-2 can be allocated to the most probable infecting variant using the Corona Array (CA). For 106 COV^-^/VAC^-^ children and adolescents with SI (CA^+^, **(A)** recruited during **(B)** Alpha, **(C)** Delta, **(D)** BA.1, **(E)** BA.2 or **(F)** BA.4/5 waves, ratios of MFI_VoC_ to MFI_WT_ (VoC-to-WT ratio) were calculated as a surrogate marker for the detection of the most probable infecting VoC. Samples with highest antibody levels against S1_WT and therefore with VoC-to-WT ratios below 1 were classified as infected with the WT virus. Samples were assumed to be infected with a variant when the VoC-to-WT ratio was highest.

We next calculated the VoC-to-WT ratio to determine the most probable infecting VoC in children and adolescents with SI (**Figure 5**), as the immune system of COV^+^ patients was able to discriminate between the S1 allelic variants of the circulating VoCs (**Figure 3**). Since the time of infection was unknown in this cohort, probands were assigned to the VoC waves based on the date of recruitment.

Out of 106 SI identified using the CA (**Figure 4**, right panel), 47 (44.3%) exhibited the highest VoC-to-WT ratio for the Alpha variant, indicating an infection with this variant (**Figure 5A**). For WT, Delta and Omicron variants BA.1, BA.2 and BA.4/5, these proportions were lower (n=5 (4.7%), n=10 (9.4%), n=15 (14.2%), n=18 (17.0%), and n=11 (10.4%), respectively).

During the Alpha wave, 16 of 26 CA^+^ subjects exhibited the highest VoC-to-WT antibody ratio for S1_Alpha (**Figure 5B**), suggesting that they were indeed in contact with the Alpha variant of SARS-CoV-2. It was unexpected to observe the highest VoC-to-WT antibody ratio for the BA.1 or BA.4/5 S1 domains in eight children recruited during the Alpha wave, despite the fact that these VoCs were not circulating at that time. The majority of these samples, however, had S1_BA.4/5 antibody levels just above the cut-off and displayed no reactivity to the other tested SARS-CoV-2 S1 antigens (data not shown). This may have caused a distortion of the VoC-to-WT ratios.

The majority of subjects recruited during the Delta wave (n=26) were most likely exposed to the Alpha variant (n=18), followed by the Delta variant (n=4), the WT strain (n=3), and the BA.1 variant (n=1) (**Figure 5C**). For subjects recruited during the Omicron waves, S1 antibody signatures pointed to infecting variants that again matched the currently or previously prevailing variants (**Figures 5D–F**).

The VoC-specific antibody profiling of children with SI often indicated the prevailing SARS-CoV-2 variants as the likely cause of infection. However, as expected, the correlation between antibody binding and recruitment into the study (COV^-^) was less stringent than that between antibody binding and known time point of infection (COV^+^).

### 3.7 Seroconversion to endemic HCoVs occurs in early childhood and was not affected by the SARS-CoV-2 pandemic

**Figure 6:**
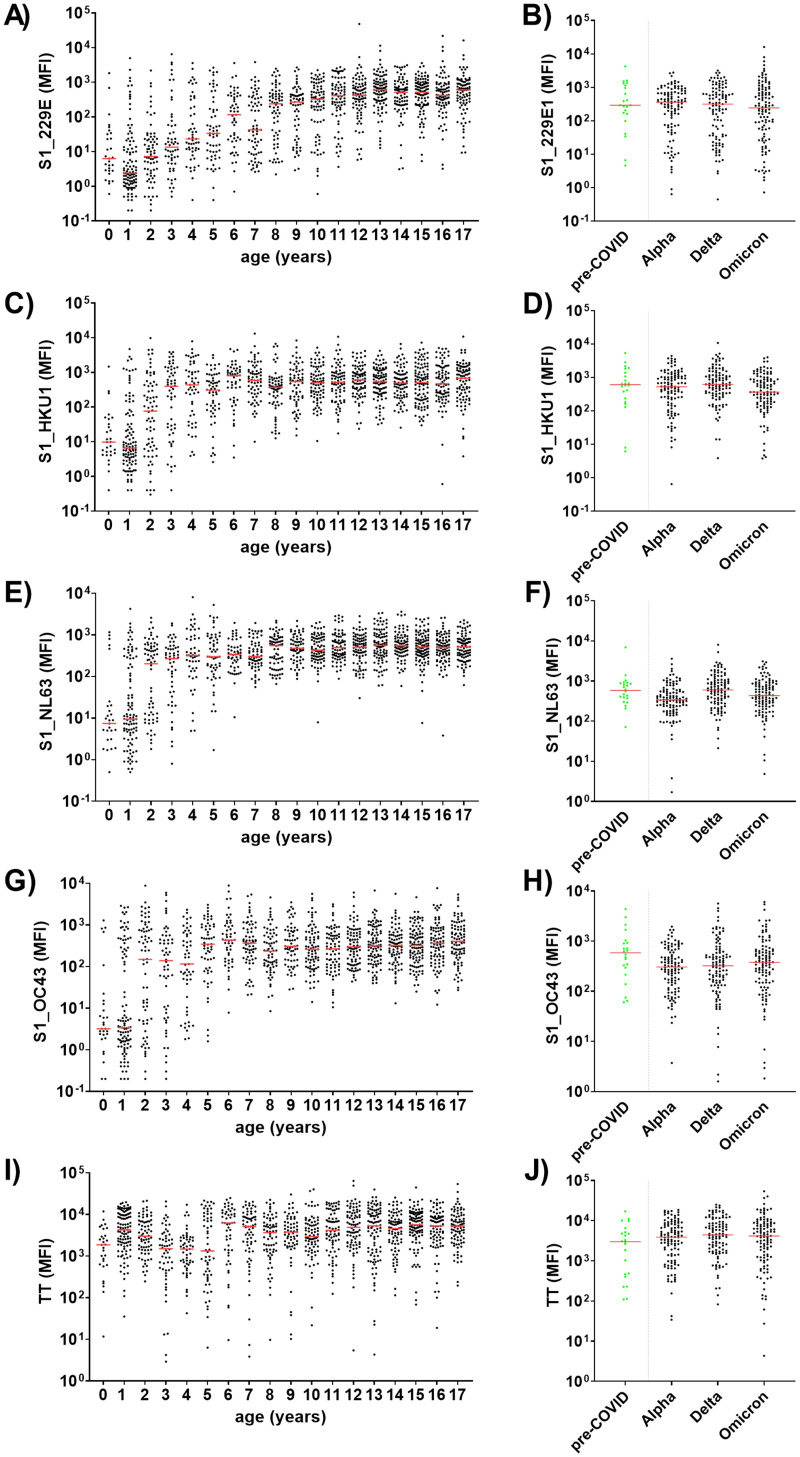
HCoV-specific antibody levels are age-dependent and are not affected by the SARS-CoV-2 pandemic. Age-dependent serum IgG levels were determined against the S1 domain of the four endemic HCoVs 229E **(A)**, HKU1 **(C)**, NL63 **(E)** and OC43 **(G)**, as well as tetanus toxoid (TT) as positive control **(I)** among 1,309 children and adolescents recruited during the SARS-CoV-2 pandemic. HCoV-specific antibody levels from pre-COVID (n=22; green) children and adolescents (median age 12 years) were compared to age-matched samples from three SARS-CoV-2 waves **(B, D, F, H)**. Matching was conducted regarding age and study site (all samples were from the University Medicine Greifswald, Germany): for each pre-COVID control five samples from one of three defined time frames (Alpha, Delta or Omicron) were matched (1:5 ratio). Significance between pre-COVID and Alpha, Delta or Omicron waves was tested with Kruskall-Wallis test with *post-hoc* Dunn´s correction. Median values are shown in red.

One of the objectives of this study was to assess whether the SARS-CoV-2 pandemic altered the seroconversion rate to endemic HCoVs in children and adolescents. To this end, antibodies against the S1 domains of the four endemic HCoVs, namely 229E, HKU1, NL63 and OC43, were measured in 1,309 children and adolescents. As a positive control, antibodies against the recall antigen tetanus toxoid were also measured (**Figure 6**). For all four HCoVs, seroconversion occurred during early childhood. Antibody levels increased sharply in the first years of life and plateaued at approximately eight years for 229E, four years for HCoV-HKU1 and NL63, and five years for OC43 (**Figures 6A, C, E, G**, respectively). The kinetics of anti-TT antibodies reflected the German vaccination recommendations, which include a baseline vaccination at two months of age and booster vaccinations at an age of 5 – 6 years (29). This resulted in a first antibody peak at 1 year of age and another steep increase around the age of six years (**Figure 6I**).

To ascertain the impact of the hygiene measures implemented during the SARS-CoV-2 pandemic on antibody titers to HCoVs in our cohort, we compared anti-HCoV-S1 antibody levels during a pre-pandemic period with an age-adjusted subsample of the COVIDKID cohort. Our findings revealed no significant differences throughout the pandemic waves in comparison to pre-pandemic levels (**Figures 6B, D, F, H**).

**Figure 7:**
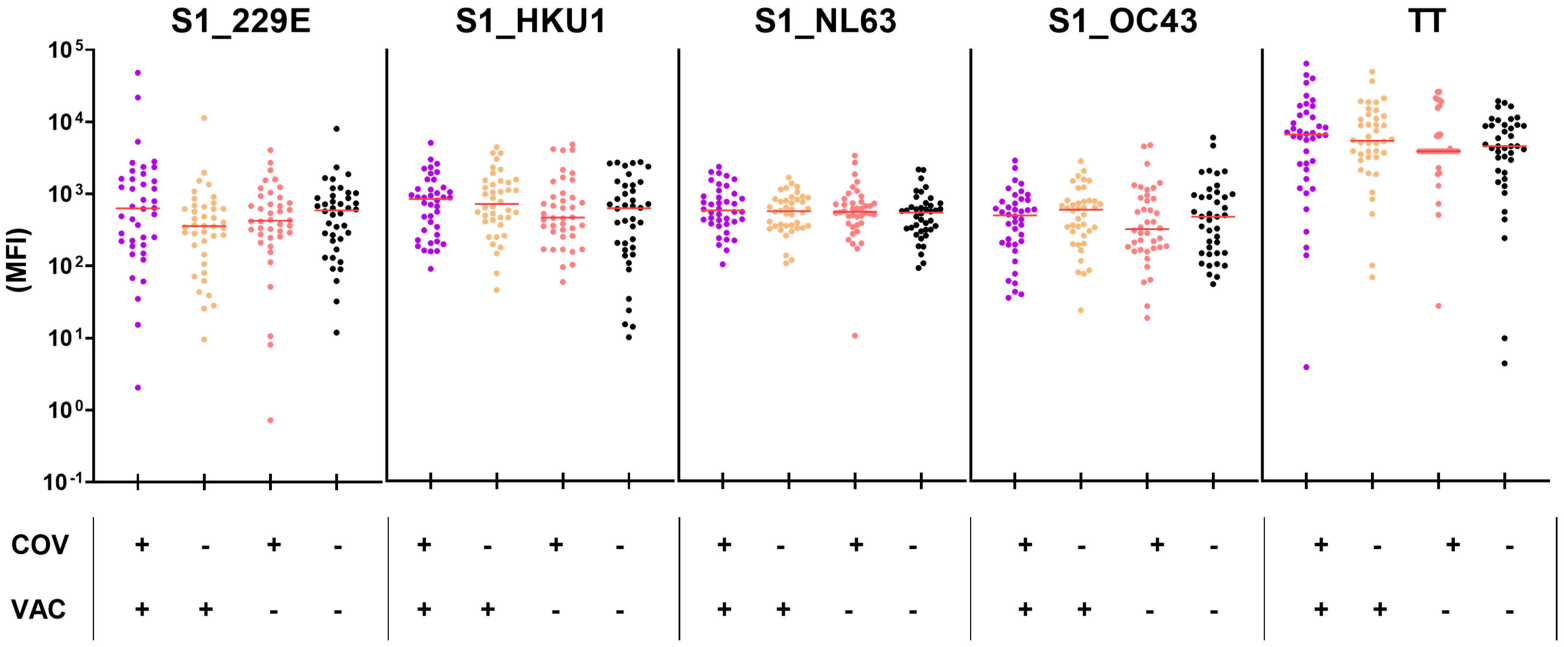
Neither SARS-CoV-2-infection nor vaccination elicits higher titers for HCoV-S1 domains in children and adolescents. Anti-HCoV-S1 antibody levels were compared in naïve (COV^-^/VAC^-^), COV^+^ and/or VAC^+^ children and adolescents (n=40 per group, matched by age and time of study inclusion in a 1:1 ratio). Antibodies against tetanus toxoid (TT) were included as control. The median is shown in red. Significance between all four groups was tested by Kruskall-Wallis test with *post-hoc* Dunn´s correction.

Finally, to investigate whether vaccination or infection with SARS-CoV-2 induces or enhances HCoV-cross-reactive antibodies, we also compared HCoV-S1-specific antibody levels in naive vs. SARS-CoV-2-exposed (VAC^+^ and/or COV^+^) children and adolescents, matched by age and time of study inclusion (median age 10 and 12 years, respectively). Neither VAC^+^ nor COV^+^ children exhibited higher HCoV-S1-specific antibody levels compared to the naïve children (COV^-^/VAC^-^; seronegative for SARS-CoV-2 S1) (**Figure 7**), suggesting that the SARS-CoV-2 serostatus had no influence on HCoV-S1-specific antibody titers in this age stratum.

In conclusion, seroconversion to endemic HCoVs occurs in early childhood and, in terms of S1-specific antibodies, was not affected by the SARS-CoV-2 pandemic.

## 4 Discussion

Seroconversion to circulating SARS-CoV-2 variants can provide valuable information for SARS-CoV-2 surveillance. In this study, we performed extensive anti-SARS-CoV-2 antibody profiling in more than 1,300 children and adolescents screened between December 2020 and March 2023, covering several SARS-CoV-2 waves. Both SARS-CoV-2 infection and vaccination induced high levels of specific antibodies against the S1 domains from SARS-CoV-2 WT and VoCs. The antibody profiles against the spike S1 domain from WT and VoCs were highly discriminatory and reflected the kinetics of VoC waves in the study region. Furthermore, our highly sensitive Luminex^®^-based approach discovered more SI than a conventional ELISA and, additionally, provided hints at the infecting VoC. Finally, vaccination or infection with SARS-CoV-2 did not induce HCoV S1-cross-reactive antibodies in children and adolescents.

Our in-house, Luminex^®^-based CA exhibited a higher sensitivity and a broader detection range than commercial ELISA kits. The assay sensitivity for anti-S1 antibodies increased from 61% (ELISA) to 93% (CA), resulting in a reduction in false-negative cases of SARS-CoV-2 infections and, conversely, a 1.43-fold increase in correctly identified COV^+^ cases. Similarly, other research groups have reported a sensitivity of 98% for a Luminex^®^-based SARS-CoV-2 6-plex for an adult cohort (30).

Moreover, the CA was more suitable for detecting SI in COV^-^ children and adolescents, with detection rates of 12.1% for CA as compared to 6.3% using the commercial ELISA. Two other studies conducted in Germany during the initial SARS-CoV-2 waves (2020 – 2021) reported lower rates of seroconversion. Specifically, 0.87% of the total cohort for the year 2020 (13) and 4.4% of children without SARS-CoV-2 infections in 2020 – 2021 were seropositive (12). As our study recruited subjects up until March 2023, it is reasonable to anticipate a higher seroprevalence. The ratio of seropositive cases (CA-based) to diagnosed and recalled infections was 1.43 (301 CA-positive cases/210 COV^+^/VAC^-^) in our study. The proportion of silent infection was considerably higher in earlier studies, with rates of 3.9 (12) and 6 (13), respectively. This highlights the significant advancement in the clinical diagnosis of COVID-19 during the pandemic.

Luminex^®^-based seroprevalence studies can serve as an invaluable component of SARS-CoV-2 surveillance. Despite the fact that SARS-CoV-2 has now entered the endemic phase (31), monitoring the humoral immunity to SARS-CoV-2 remains vital not only to gain insights into the impact of circulating VoCs, but also to monitor waning immunity, identify immune escape variants and inform vaccination programs. Given that vaccination against COVID-19 is no longer recommended for healthy children in Germany (32), it is likely that primary SARS-CoV-2 infections will occur at an early age. In this endemic scenario, monitoring the S1 seroprevalence will be the most informative marker for SARS-CoV-2 exposure in infants and children. The Luminex^®^-based approach can be utilized to monitor the kinetics of SARS-CoV-2 infection in infants and children, with the objective of determining whether they will converge with those of endemic HCoVs, which plateau at approximately five to six years of age (as discussed below). In addition to its implications for the current pandemic, our Luminex^®^-based approach offers an efficient and easily expandable tool for the surveillance of future outbreaks with other pathogens.

As both COVID-19 vaccination and SARS-CoV-2 infection induce S1-specific antibodies, we investigated whether seroconversion to NC could serve as a reliable marker for previous COVID-19 infection(s). However, anti-NC antibodies alone were not sufficient to reliably detect (silent) SARS-CoV-2 infections in children and adolescents. In contrast to the S1 antigen, the CA did not improve the sensitivity for the detection of anti-NC antibodies (ELISA: 49%; CA: 44%). Conversely, the assay specificity for both the CA and ELISA was 96%. Consequently, a positive NC test result can be taken as an indication of an (unnoticed) infection.

The low assay sensitivity can be attributed to the presence of low-level, cross-reactive anti-NC antibodies in children and adolescents who had not been exposed to SARS-CoV-2, exemplified by our pre-pandemic cohort (22, 23, 27, 33). This basal antibody binding required a much higher cut-off value (NC: 545.7; S1_WT: 50.4), which reduced sensitivity. Nevertheless, higher sensitivities might be achieved with different detection systems, such as the double-antigen sandwich assay format utilized in Elecsys^®^ anti-SARS-CoV-2 immunoassays (sensitivity: 97.8%; specificity: 98.5%) (34). Another reason for the lower sensitivity of NC versus S1 antibody detection systems could be the relatively swift decay of NC- versus S-specific antibody levels (35). Moreover, the immune response of children to SARS-CoV-2 infection is predominantly directed towards the S protein, rather than the NC (14). Consequently, the absence of anti-NC antibodies is not suitable to exclude previous SARS-CoV-2 infection in children and adolescents.

The results of this study clearly demonstrated that the S1-specific antibody response can discriminate between different SARS-CoV-2 VoCs. To enhance the CA’s discriminatory power, the analysis was focused on the more variable S1 domain, rather than using the full-length S protein. Indeed, several studies have demonstrated that the more conserved S2 domain contains numerous cross-reactive epitopes, not only with other SARS-CoV-2 VoCs, but also with the closely related HCoVs (23, 27, 36, 37). The presence of cross-reactive antibodies directed at the S2 domain would have blurred our results. In contrast, antibodies cross-reacting to the RBD/S1 domain have only rarely been reported (23, 33, 36, 37).

Our multiplex approach demonstrated that antibody binding patterns for the S1 domains of SARS-CoV-2 WT and VoCs are highly individual and allele-specific. In most cases, the strongest antibody response was detected against the most likely infecting VoC, e.g. the prevailing VoC at the time of diagnosis. This demonstrates that in terms of the spike S1/RBD domain, the antibody response is highly VoC-specific, as previously reported (16, 27, 33, 38, 39). However, in each SARS-CoV-2 wave we also observed subjects that showed a specific antibody response to other VoCs. This is not unexpected and actually reflects the pandemic situation in Northern Germany with several variants circulating in parallel (3; personal communication Meyer-Bahlburg). Consequently, the S1-specific antibody profiling can provide insights into the causative VoC.

To the best of our knowledge, allele-specific antibody profiles have not been investigated on a large scale until now. Some studies have conducted RBD-specific antibody profiling and performed correlation analyses for comparing antibody binding to WT versus Alpha, Beta and Delta variants. These studies demonstrated comparable IgG binding to RBD from both WT and Alpha variant, but reduced binding to RBD from the Beta variant in children and/or adults recruited in the first wave of the pandemic (16, 38). The Omicron variant exhibits an even more pronounced immune escape, as evidenced by the markedly reduced antibody binding to the Omicron Spike protein in vaccinated individuals (17, 18). Our study is distinctive in two ways: Firstly, the study spanned a considerable period of time (December 2020 – March 2023), allowing the subjects to be allocated to six distinct SARS-CoV-2 waves (WT, Alpha, Delta, and Omicron BA.1, BA.2 and BA.4/5). Secondly, the conversion of S1 domain-specific antibody responses into VoC-to-WT ratios enabled a comparison of antibody profiles against all VoCs on an individual level. This approach proved to be a robust tool for estimating exposure to SARS-CoV-2 on a variant-specific level.

Furthermore, S1 variant-specific serological signatures can also provide insights into the infecting VoC in silent SARS-CoV-2 infections. In our study cohort, 12.1% of children and adolescents experienced SI, as determined by an antibody response against the S1 domain from the WT virus or VoCs. We again employed the S1 variant-specific antibody signatures to determine the most likely infecting VoC in these children. Since the time of infection is unknown in this cohort, probands were assigned to the VoC waves based on the date of recruitment. Consequently, they might have been infected with the prevailing VoC or previously circulating variants, which is clearly reflected in our data. For instance, children and adolescents recruited during the Delta wave showed the highest antibody binding for S1 from the Alpha or Delta VoCs, but not from BA.2 and BA.4/5. The majority of detected SI presented with an antibody signature for the Alpha variant, which dominated in North-Eastern Germany between December 2020 and June 2021. Overall, S1-variant specific antibody signatures can provide valuable insights into the contact variant in children with SI. However, it should be noted that the reliability of our analysis is contingent upon the absence of a prior SARS-CoV-2 vaccination or infection, as both of these conditions induce high titers of SARS-CoV-2 S1-specific antibodies.

Endemic HCoVs are a common cause of acute respiratory infections, leading to a wide range of disease severity, especially during the winter months. A meta-analysis attributed 5.9% (range: 0.9 – 18.4%) of respiratory infections in children to HCoVs on a global scale (19). In order to gain further insight into the epidemiology of these viruses, we profiled the antibody binding to the S1 domain of endemic HCoVs. Our findings confirmed that seroconversion occurs in early childhood. The antibody levels against HCoV-S1 domains exhibited a marked increase with age, reaching a plateau at approximately four to eight years of age. This pattern aligns with the findings of other seroprevalence studies in Germany and France, which reported that many infants experienced HCoV infections in their first two years of life (16, 20).

Remarkably, seroconversion to endemic HCoVs was not affected by the SARS-CoV-2 pandemic, despite reduced contact rates and implemented hygiene measures. However, our pandemic and pre-pandemic cohorts consisted primarily of older participants (median age of 12 and 10 years, respectively). This limits the sensitivity for the discovery of changes caused by the pandemic, as anti-HCoV antibody levels had already plateaued at 4-8 years of age. Indeed, Sikkema et al. observed a reduction in S1 seroprevalence during the SARS-CoV-2 pandemic only in very young Dutch children (aged <1 year, for all four HCoVs), and only for NL63 also in older age groups (up to 18 years) (40).

Finally, we investigated whether vaccination or infection with SARS-CoV-2 induces HCoV-cross-reactive antibodies, focusing our analysis on the highly variable spike S1 domain. Our data demonstrated that there was no increase in antibodies specific to the S1 domain of HCoVs following SARS-CoV-2 infection or vaccination. Other research groups employed the full-length spike protein or the S2 domain, and reported elevated HCoV antibodies (particularly against OC43 and HKU1) post-infection and -vaccination (36, 41, 42). Consequently, infection and vaccination elicit cross-reactive antibodies, although these antibodies predominantly recognize epitopes on the more conserved S2 domain.

A limitation of our study is the use of a single dilution for plasma samples for the CA. Indeed, serum/plasma titrations are in general a more accurate approach to obtain quantitative information on antibody reactivity (27, 43). For incorporation into the clinical routine, however, diagnostic tests should ideally provide information from a single serum/plasma dilution, thus enabling sufficient throughput. This is the reason why a significant number of published studies using Luminex-based assays employ a single dilution, ranging from 1:100 to 1:2000 (44–47). In our study, a 1:10,000 plasma dilution was identified as the optimal dilution for our semi-quantitative readout, enabling accurate detection by avoiding saturation effects at high antibody levels, while ensuring the proper detection of low antibody levels in the majority of cases. Given the relatively large sample size of 1,309 subjects in this study, this approach enabled the conservation of reagents and time, while maintaining data quality. The larger dynamic range of the CA compared to the ELISA approach proved advantageous in this context.

In conclusion, our highly sensitive Luminex^®^-based Corona Array represents a valuable tool for monitoring the S1-specific antibody response against SARS-CoV-2 WT and VoCs as well as HCoVs in children and adolescents, and has the potential to serve as a surveillance tool. By focusing on the variable S1 domain, we were able to identify the most likely SARS-CoV-2 contact variant in children with diagnosed and silent infections. This has opened up new possibilities for addressing underreporting during pandemics.

## Data Availability

The raw data supporting the conclusions of this article will be made available by the authors, without undue reservation.
