# Supplementary material for: Variant-specific antibody profiling for tracking SARS-CoV-2 variant infections in children and adolescents
Source: Front Immunol. 2024 Aug 27;15:1434291. doi: 10.3389/fimmu.2024.1434291 (PMC11384586; doi:10.3389/fimmu.2024.1434291)
Supplement: Supplementary file 1 [file DataSheet1.pdf]

frontiers

Supplementary Material

1.1 Supplementary Figures

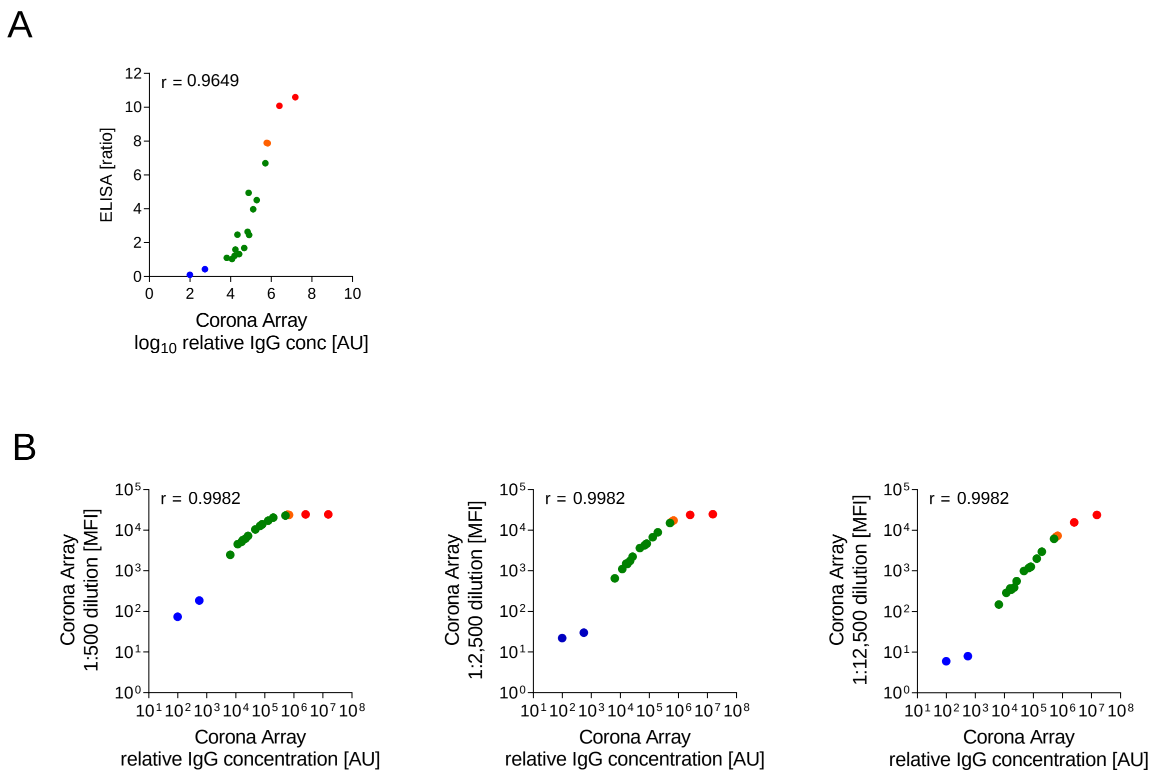

Supplementary Figure 1. Determination of a single dilution for Corona Array (CA) analyses. 19 plasma samples with no (n=2, blue), average (n=13, green), high (n=2, orange) and very high (n=2, red) anti-S1 WT IgG levels, as determined by a commercial S1 IgG ELISA, were titrated over a wide range (1:20 – 1:325,000) and analyzed in the CA. The relative IgG concentration was calculated based on a non-linear regression model (28). A) Correlation of log10-transformed relative IgG concentrations (CA) with values obtained with a commercial S1 IgG ELISA. The dynamic range of the dilution-based CA is much larger than for the ELISA (5 versus 2 orders of magnitude). B) Correlation of raw MFI values obtained from a single dilution with relative IgG concentrations (dilution series). Spearman´s correlation coefficient r is shown. The IgG levels obtained from single-point dilutions were strongly correlated (r>0.99) with the dilution-based IgG levels, even though the dynamic range slightly decreased (ca. 4 logs). Based on these results, a 1:10,000 dilution of plasma samples was chosen for the CA analyses.
